# *VaERD15*, a Transcription Factor Gene Associated with Cold-Tolerance in Chinese Wild *Vitis amurensis*

**DOI:** 10.3389/fpls.2017.00297

**Published:** 2017-03-07

**Authors:** Dongdong Yu, Lihua Zhang, Kai Zhao, Ruxuan Niu, Huan Zhai, Jianxia Zhang

**Affiliations:** ^1^College of Horticulture, Northwest A&F UniversityYangling, China; ^2^Key Laboratory of Horticultural Plant Biology and Germplasm Innovation in Northwest China, Ministry of AgricultureYangling, China; ^3^State Key Laboratory of Crop Stress Biology in Arid Areas, Northwest A&F UniversityYangling, China

**Keywords:** grapevine, *Vitis amurensis*, *VaERD15*, cold tolerance, functional analysis

## Abstract

Early responsive to dehydration (*ERD*) genes can be rapidly induced to counteract abiotic stresses, such as drought, low temperatures or high salinities. Here, we report on an *ERD* gene (*VaERD15*) related to cold tolerance from Chinese wild *Vitis amurensis* accession ‘Heilongjiang seedling’. The full-length *VaERD15* cDNA is 685 bp, including a 66 bp 5′-untranslated region (UTR), a 196 bp 3′-UTR region and a 423 bp open reading frame encoding 140 amino acids. The VaERD15 protein shares a high amino acid sequence similarity with ERD15 of *Arabidopsis thaliana*. In our study, *VaERD15* was shown to have a nucleic localization function and a transcriptional activation function. Semi-quantitative PCR and Western blot analyses showed that *VaERD15* was constitutively expressed in young leaves, stems and roots of *V. amurensis* accession ‘Heilongjiang seedling’ plants, and expression levels increased after low-temperature treatment. We also generated a transgenic *Arabidopsis* Col-0 line that over-expressed *VaERD15* and carried out a cold-treatment assay. Real-time quantitative PCR (qRT-PCR) and Western blot analyses showed that as the duration of cold treatment increased, the expression of both gene and protein levels increased continuously in the transgenic plants, while almost no expression was detected in the wild type *Arabidopsis*. Moreover, the plants that over-expressed *VaERD15* showed higher cold tolerance and accumulation of proline, soluble sugars, proteins, malondialdehyde and three antioxidases (superoxide dismutase, peroxidase, and catalase). Lower levels of relative ion leakage also occurred under cold stress. Taken together, our results indicate that the transcription factor *VaERD15* was induced by cold stress and was able to enhance cold tolerance.

## Introduction

Grapevine (*Vitis vinifera* L.) is one of the most important multi-use fruiting plants of the world, and its berries are used in wine-making, or eaten fresh or dried. In addition to their value as a food (providing sugars, roughage etc.), grapes and their processed products have also been shown to have nutraceutical benefits including those attributed to resveratrol, which has a role in preventing and treating cardiovascular disease and cancer (Jeandet et al., 1995; Yang et al., 2009). These beneficial properties have fostered the recent growth in the grape industry.

The majority of commercial grape cultivars belong to the European grape (*V. vinifera* L.). While these cultivars have excellent organoleptic qualities, they suffer relatively poor tolerance to the cold experienced during winter, resulting in significant damage to grapevines growing in the cooler regions of the world, including northern America and northern China.

China has abundant resources of wild grape germplasm. These include *V. amurensis*, a species that can tolerate very low winter temperatures approaching -32°C. Therefore, this species has great potential as a germplasm resource for cold-resistant breeding (He and Niu, 1989). Study of the cold-tolerance genes in *V. amurensis* has significantly contributed to understanding the mechanisms of cold-tolerance, as well as transgenic breeding (Xu et al., 2014a,b).

In *Arabidopsis*, *ERD* genes can be induced within 1 h by drought stress (Kiyosue et al., 1994). Kiyosue et al. (1994) divided a total of 26 *ERD* cDNA clones into 16 different gene families based on their expression in response to drought. Early research on *ERDs* was concentrated on *Arabidopsis*, and many studies have shown that *ERD* genes can be induced by a diversity of stresses including drought (Rai et al., 2012), low temperature (Kiyosue et al., 1998), salinity (Rai et al., 2015) and abscisic acid (ABA) (Aalto et al., 2012). In recent years, *ERD* genes have been isolated from corn (Liu et al., 2009), soybeans (Alves et al., 2011a), tobacco (Shao et al., 2014), and tomato (Ziaf et al., 2016). Importantly, their roles in response to a range of stresses have been identified. However, of these *ERD*s, only *ERD6*, *ERD10*, and *ERD15* are related to cold tolerance in *Arabidopsis*. The first of these, *ERD6*, encodes a sugar carrier protein and is induced by low temperatures and moisture stress (Kiyosue et al., 1998). The gene *AtERD10* is induced by cold and regulates *CBF* transcription factors, and the transfer DNA (T-DNA) insertion silence strain of *AtERD10* reduces stress tolerance of mutants compared with wild type *Arabidopsis* plants (Kim and Nam, 2010). An earlier report indicates that, at low temperatures, *SpERD15* can protect the cell membrane, improve photosynthetic efficiency and promote the accumulation of soluble substances (Ziaf et al., 2011). However, another report (Kariola et al., 2006) showed that over-expression of *AtERD15* decreased the sensitivity to ABA, tolerance to drought, and also to low temperatures. Conversely, the *AtERD15* mutant was more sensitive to ABA and enhanced the tolerance to salinity and drought (Kariola et al., 2006). Therefore, in different plants, *ERD15* may have different functions. There have been no previous reports on the *ERD* genes in Chinese wild *Vitis* spp., and for this reason we are keen to explore the role of *ERD15* in *V. amurensis*.

We have already confirmed that *V. amurensis* is the most cold-resistant species of the 18 wild grapes species native to China and the seven wild grapes species native to North America (Zhang et al., 2012). Subsequently, a cold-induced cDNA library was constructed using potted plants of *V. amurensis* accession ‘Heilongjiang seedling’, in which one EST sequence encoding the ERD protein was obtained, and the gene was named as *VaERD15*. Real-time quantitative PCR (qRT-PCR) analyses revealed that *VaERD15* was induced by cold stress (Zhang et al., 2013). In this study, we clone the full-length *VaERD15*, and confirm its function in response to cold stress.

## Materials and Methods

### Plant Materials and Growth Conditions

Plants of Chinese wild *V. amurensis* accession ‘Heilongjiang Seedling’, which originates in northeast China, were maintained in the grape germplasm repository at the Northwest A & F University, Yangling, Shaanxi, the People’s Republic of China. This accession is highly resistant to cold (Zhang et al., 2012).

In early January 2010, 1-year-old shoots were taken from mature vines for sand-storage at under 4°C. At the end of March, the shoots were retrieved and used for cuttings, soaked for 2 h in Transplantone (500 mg/L), allowed to develop roots, and cultivated in a greenhouse (25°C, light 12000 lux). In July, well-grown and healthy potted plants were selected for further cold stress and total RNA and protein isolation.

### Isolation and Sequence Analysis of the *VaERD15* Gene

‘Heilongjiang seedling’ plants growing in pots were placed in a pre-chilled growth chamber at 4°C. Young leaves, stems and roots were harvested at 0, 2, 6, 12, 24, and 48 h after exposure to 4°C. Samples were frozen in liquid nitrogen prior to extraction of RNA and protein.

Total RNA was extracted using the improved sodium dodecyl sulfate (SDS)/phenol method (Zhang et al., 2003). The first-strand cDNA was synthesized using the Easy Script First-Strand cDNA Synthesis Super Mix (Transgen, China), according to the manufacturer’s protocol. The cDNA templates for 5′- and 3′-Rapid Amplification of cDNA Ends (RACE) were synthesized using the SMARTTM RACE cDNA Amplification Kit (Clontech, Palo Alto, CA, USA). Primers for 5′- and 3′-RACE are listed in **Table 1**. All amplified RACE fragments were sequenced three times for each sample. Based on the 5′-RACE and 3′-RACE results, a pair of full-length primers *VaERD15*-F/R (including initiation and termination codons) were designed (**Table 1**). Amplification and sequencing of the full-length cDNA of *VaERD15* were repeated using two replicates.

**Table 1:** Primers used in this study.

Sr. no.	Primer	Sequence	Amplicon (bp)
1	*VaERD15-3′*RACE	5′-GTTCGTTCCGTTGGCATATCGGACGGT-3′	
	*VaERD15-5′*RACE	5′-CTCAAACTACCAGGAAACCCTAACTCGC-3′	
2	*VaERD15-*F(rt *VaERD15* -F)	5′-ATGGCTATGGAGGTAATTTCACGTAC -3′	423
	*VaERD15-*R(rt *VaERD15* -R)	5′- TTACCGCGGCTGCTGAATCG -3′.	
3	*VaERD15-* XbalI-F	5′-GCTCTAGAATGGCTATGGAGGTAATTTCACG-3′	439
	*VaERD15-*Kpn I -R	5′-GGGGTACCCCGCGGCTGCTGAATC-3′	
4	VaERD15-NcoI -F(Southern)	5′-CTAGccatgg ATGGCTATGGAGGTAATTTCACG-3′	442
	*VaERD15-Bglll* -R	5′-GGAagatetCTACGGCGGCTGCTGAATCG-3′	
5	*Actin1-F*	5′-GATTCTGGTGATGGTGTGAGT-3′	168
	*Actin1-R*	5′-GACAATTTCCCGTTC AGC AGT-3′	
6	*AtGAPDH-F*	5′-TTGGTGAC AAC AGGTC AAGC A-3′	
	*AtGAPDH-R*	5′-AAACTTGTCGCTCAATGCAATC-3′	
7	*rt AtERD15-F*	5′-AACTTCGACTTGGTACCCTGAT-3′	116
	*rt AtERD15 -R*	5′-GAAGATCAGCTACATCGATATGA-3′	


The cDNA sequence of *VaERD15* gene from *V. amurensis* was translated into amino acid sequences on the NCBI website^1^. The protein conserved domain analysis website^2^ was used to predict conserved domains, theoretical molecular weights and isoelectric points. The homologies of *ERD15* in *V. amurensis* and in corn, pepper and *Arabidopsis* were analyzed by means of DNAMAN analysis software. Finally, phylogenetic analyses were generated using MEGA 5.0 software^3^.

### Expression Pattern Analysis of *VaERD15*

Total RNA was extracted from various grapevines after exposure to low-temperature stress (4°C) for 0, 2, 6, 12, 24, and 48 h, and first-strand cDNA was synthesized, as previously described. Semi-quantitative PCR was carried out using the primers rt*VaERD15*-F and rt*VaERD15*-R (**Table 1**). The volume of the semi-quantitative PCR amplification was 20 μl, and *Actin1* (accession no. AY680701) was used as the internal reference gene. All reactions were repeated for three biological replicates.

Western blotting was carried out to further analyze the expression pattern of *VaERD15*. Total protein was extracted from various samples according to the method of Méchin et al. (2006). The extracted protein concentration was determined using the method of Bradford (1976). Proteins samples (25 μg) were prepared for SDS-polyacrylamide gel electrophoresis (PAGE), and blotted onto a polyvinylidene fluoride membrane (Roche, product no. 03010040001). Immune antibodies were prepared in our laboratory, as previously reported (Zhang et al., 2014).

### Subcellular Localization of *VaERD15*

The ORF of *VaERD15* without a termination codon was obtained by PCR amplification using specific primers *VaERD15*-XbalI-F and *VaERD15*-KpnI-R (**Table 1**). The PCR products digested by XbalI and KpnI were cloned into a pMD18-T vector (TaKaRa, Japan). The fragments were fused into the N-terminus of the GFP expression vector driven by the 35S promoter. The vector carrying 35S::GFP was used as a control. The plasmids of the 35S::*VaERD15*-GFP fusion construct and 35S::GFP were purified for subsequent experiments. Plasmids were then transformed into onion epidermal cells using the particle bombardment method, as described by Varagona et al. (1992). Transformed onion epidermal cells were cultured on MS media under dark conditions for 24 h at 25°C. Expression of the genes transformed into the onion epidermal cells was observed using confocal laser scanning microscopy (LSM 510 META, ZEISS, Germany).

### Transcriptional Activation Assay

For the transcriptional activation assay, the ORF of *VaERD15* was generated and fused into the frame to the NcoI and BamHI sites of the GAL4 DNA-binding domain in the pGBKT7 vector by recombination reactions (Invitrogen, USA). The expression vector pGBKT7 carrying the *GAL4* gene constructed by our laboratory was used as a positive control and the empty pGBKT7 vector was used as a negative control. These constructs were then transformed into the yeast strain AH109. The resulting transformants were streaked onto Synthetic Defined (SD)/-Trp medium. After incubation at 30°C for 3 days, transformed strains on the SD/-Trp plates were selected and streaked onto SD/-Trp/-His/-Ade plates containing x-α-gal; the level of transcriptional activation was evaluated by color reaction.

### Generation and Detection of Transgenic *Arabidopsis* Seedlings

The full-length cDNA of *VaERD15* was amplified by PCR and cloned into the BglII/NcoI site of pCAMBIA3301, generating pCAMBIA3301-*VaERD15*. The specific primers *VaERD15*-Ncol-F/R are shown in **Table 1**. Constructs were verified by sequencing. The constructed plasmid was introduced into *Agrobacterium tumefaciens* GV3101 cells by electroporation. Transgenic *Arabidopsis* plants were obtained using the floral dipping method (Clough and Bent, 1998). Putative transgenic *Arabidopsis* plants harboring the pCAMBIA3301-*VaERD15* construct were selected on MS plates containing 10 mg/L Basta.

Homozygous T3 *Arabidopsis* strains were tested by PCR and genomic DNA was extracted from 3-week-old leaves of the putative transgenic *Arabidopsis* seedlings using the cetyltrimethyl ammonium bromide (CTAB) method with appropriate modification (Wu et al., 1998). The specific primers *VaERD15*-NcoI –F/R were used for amplifying the exogenous gene, with pCAMBIA3301-*VaERD15* plasmid DNA as a positive control, and DNA from WT *Arabidopsis* (Col-0) leaves as a negative control. At the same time, leaves of *VaERD15-*positive *Arabidopsis* plants were taken for GUS staining, with WT *Arabidopsis* plants serving as controls.

Southern blot analysis was carried out as previously described Southern (1975). After plant genomic DNA was extracted and purified, 50 μg DNA was digested by the restriction enzyme HindIII in 40 μl for 12 h. Electrophoresis and Southern blot analysis were then carried out according to the standard methods.

### Cold Tolerance, qRT-PCR and Western Blot Assay

Two T3 *Arabidopsis* lines (L1, L2) were used for the cold tolerance assay. Similarly, robust transgenic and WT 3-week-old *Arabidopsis* plants were used for the cold treatment. Plants were placed in a pre-chilled chamber at 4°C to cold acclimate for 48 h, and then transferred to a pre-chilled chamber at -6°C for 72 h. Plants suffering from cold stress were transferred to room temperature (approximately 23°C) for 5 days to recover. The phenotypic changes of *Arabidopsis* plants were observed and photographed during this period. Leaf samples were collected at 0, 2, 4, 8, 12, 24, 48, and 72 h after exposure to cold stress and stored at -80°C for qRT-PCR and Western blot analyses. qRT-PCR was carried out to determine expression changes of *VaERD15* and *AtERD15* [primers: rt*VaERD15*-F/R and rt*AtERD15*- F/R (**Table 1**)], *in vivo*, using the Takara SYBR Premix Ex Taq^TM^ II (Perfect Real Time) on a Bio-Rad IQ5 Real-Time PCR Detection System (Bio-Rad Laboratories, Hercules, CA, USA). The volume used for qRT-PCR amplification was 20 μl, and *AtGAPDH* (accession no. 101214) was used as the endogenous reference gene. Western blotting was carried out as previously described. All experiments were carried out for three biological replicates.

### Biochemical Indicator Assays of Transgenic *Arabidopsis*

Biochemical indices relating to cold stress were determined in 3-week-old *Arabidopsis* seedlings in both transgenic and control plants. All plants had been subjected to the cold treatment described above. Determinations in this part of the study were carried out for three biological replicates.

Relative electrolyte leakage was assessed according to the previously described method (Weigel et al., 2001). Proline content was determined following the method of Shan et al. (2007). MDA content was measured according to the method of Puckette et al. (2007) with minor modifications. About 200 mg leaves frozen in liquid nitrogen was homogenized in 4 ml 10% trichloracetic acid (TCA), then centrifuged at 10,000 rpm for 10 min. The supernatant (2 ml) was mixed with 2 ml thiobarbituric acid (TBA) and heated at 95°C for 30 min, quickly cooled on ice and then centrifuged at 10,000 rpm for 10 min. The soluble sugar and soluble protein contents were measured according to the methods of Bradford (1976) and Machado et al. (2013), respectively.

The activity of SOD was measured using the nitroblue tetrazolium (NBT) method (Giannopolitis and Ries, 1977; Puyang et al., 2015). POD activity was measured using the method of Pagariya et al. (2012). CAT activity was determined as described by Tseng et al. (2007).

### Statistical Analysis

All physiological data was analyzed using the IBM SPSS Statistics 18.0 software. The differences between the transgenic samples and the corresponding wild type samples were calculated by the independent sample *t*-test. Significant differences were represented by ^∗^*P* < 0.05; ^∗∗^*P* < 0.01.

## Results

### Sequence Analysis of *VaERD15*

Specific primers were designed according to the acquired ORF of *VaERD15*, which had a total length of 423 bp with a 5′-untranslated region (UTR) of 66 bp, a 3′-UTR of 196 bp and an intron size of 88 bp. *VaERD15* encoded a predicted polypeptide of 140 amino acids with a molecular weight of 16.2 kD (Zhang et al., 2014), and pI of 4.80. Alignment analysis with the grapevine genome (Jaillon et al., 2007) showed that *VaERD15* was initially located on chromosome 13.

**FIGURE 1:**
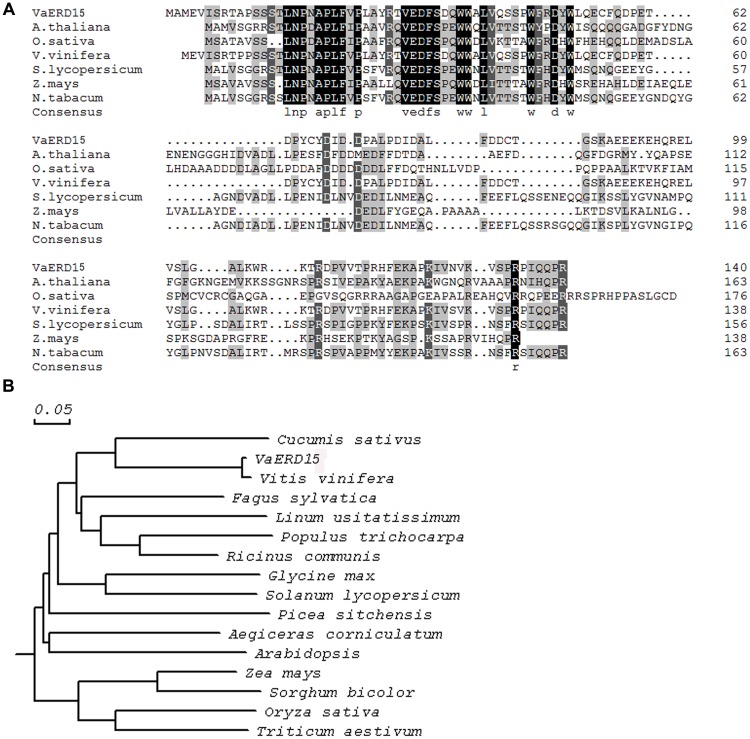
**(A)** Amino acid sequence alignment of VaERD15 and other ERD15 from similar plant species. **(B)** Phylogenetic analysis of VaERD15 and other ERD15 from similar plant species. Sequences are from *Vitis amurensis* (*VaERD15*, JQ687321), *Arabidopsis thaliana* (*AtERD15*, AAM64638.1), *Brassica napus* (*BnERD15*, ADP37978.1), *Solanum lycopersicum* (*SlERD15*, NP_001234461), *Zea mays* (*ZmERD15*, ACG25626.1), and *Capsicum annuum* (*CaERD15*, ABB89735.1).

Multiple sequence alignment analysis (**Figure 1A**) showed the *VaERD15* shared 36% homology with the *ZmERD15* predicted protein (accession no. ACG25626.1), 35% homology with the *CaERD15* predicted protein (accession no. ABB89735.1) and 33% homology with the *AtERD15* predicted protein (accession no. AAM64638.1). Phylogenetic analyses (**Figure 1B**) indicated that the relationship of *VaERD15* with other *ERDs* from similar plant species can be divided into two families. The closest relationship with *VaERD15* was found in *Vitis vinifera* and *Cucumis sativus*.

### Expression Pattern Analysis of *VaERD15*

**FIGURE 2:**
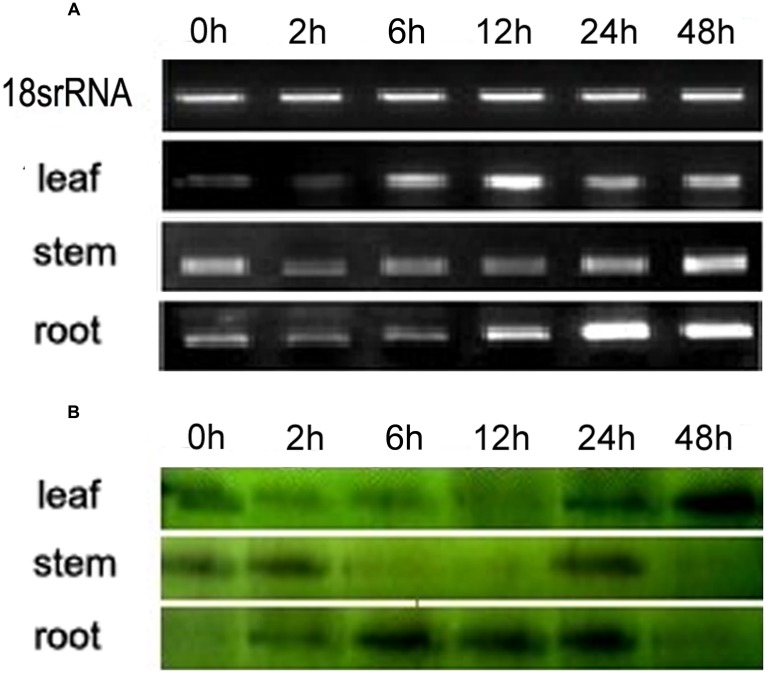
**Expression pattern analyses of *VaERD15* in different tissues of *Vitis amurensis* accession ‘Heilongjiang seedling’ after different periods of cold stress at 4°C.** **(A)** Semi-quantitative PCR was used to analyze the transcriptional level expression of *VaERD15* with *Actin1* as a reference gene. **(B)** Western blotting was carried out to detect the expression levels of VaERD15 protein after cold stress. All experiments were carried out for three biological replicates.

Semi-quantitative PCR and Western blotting were carried out to determine the expression patterns of *VaERD15*. The relative expressions at transcription level are shown in **Figure 2A**; the transcript was detected in all tissues measured. At 0 h, high *VaERD15* expression was found in stems, low expression in leaves and zero expression in roots. After 2 h of cold stress, the expression of *VaERD15* in stems decreased significantly, but recovered slightly after 12 h. The expression of *VaERD15* in leaves and roots showed a rising trend that peaked at 24 h, but then decreased.

The Western blot analysis results are shown in **Figure 2B**. We observed 16.2 kD bands on the polyvinylidene difluoride, membrane, indicating that the tissue proteins in roots, stems and leaves of *V. amurensis* accession ‘Heilongjiang seedling’ specifically reacted with *VaERD15* antibodies. *VaERD15* expression in leaves increased gradually and peaked at 48 h under the cold stress treatment. *VaERD15* expression in stems showed a downward trend up to 12 h, which recovered at 24 h, but subsequently decreased. In the roots, *VaERD15* expression levels increased, with a maximum at 6 h and a second small peak after 24 h.

### *VaERD15* Functions as a Transcription Factor

**FIGURE 3:**
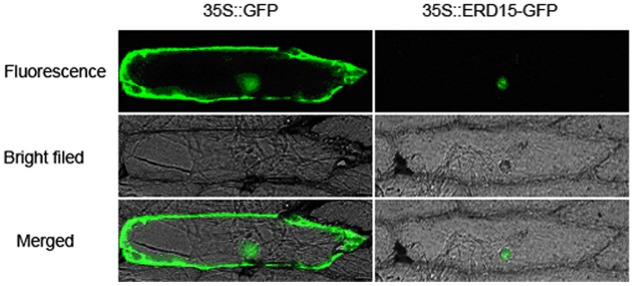
**Subcellular localization of 35S::*VaERD15*-GFP fusion protein and control in onion epidermal cells.** Cells were observed using fluorescent microscopy.

The subcellular localization results showed that the *35S: VaERD15-GFP* fusion expression vector was transiently expressed in onion epidermal cells. Green fluorescence was only observed in the nuclei, while in the control green fluorescence was visible throughout the entire onion cell (**Figure 3**). This indicates that *VaERD15* is localized to the nuclei.

**FIGURE 4:**
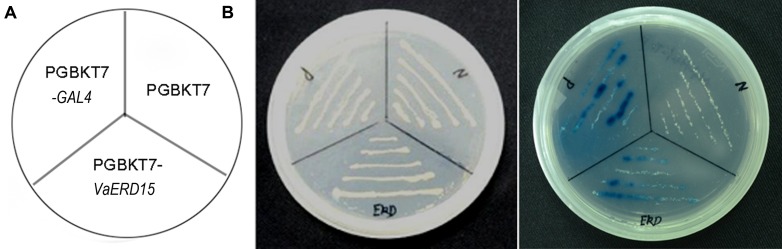
**Transcriptional activation assay of *VaERD15* in yeast.** **(A)** Fusion proteins of pGBKT7-*GAL4* (positive control), pGBKT7-*VaERD15* and pGBKT7 vector (negative control) were transformed into the yeast strain AH109. **(B)** Transformants were streaked onto plates of SD/Trp- and SD/Trp-/His-/Ade- + X-α-gal to detect their growth conditions and β-galactosidase activity.

The AH109 strains with the recombinant plasmid of pGBKT7-*GAL4* (positive control) and pGBKT7-*VaERD15* or the empty pGBKT7 vector (negative control) were all able to grow well on the SD/-Trp medium (**Figure 4A**). This demonstrates that the pGBKT7-*VaERD15* recombinant plasmid, and the positive and negative controls were all transferred into the yeast. The strains transformed with *VaERD15* were able to grow well on the SD/-Trp/-His/-Ade + X-α-gal selective medium, and turned blue on the SD/-Trp/-His/-Ade + X-α-gal medium. Accordingly, the negative control did not grow on the SD/-Trp-His-Ade medium (**Figure 4B**), indicating that *VaERD15* could activate the expression of the reporter gene and synthesize the histidine and adenine required for the normal growth of yeast AH109. Taken together, these results illustrate that *VaERD15* could function as a transcriptional activator in yeast.

### Molecular Detection of Transgenic Plants

**FIGURE 5:**
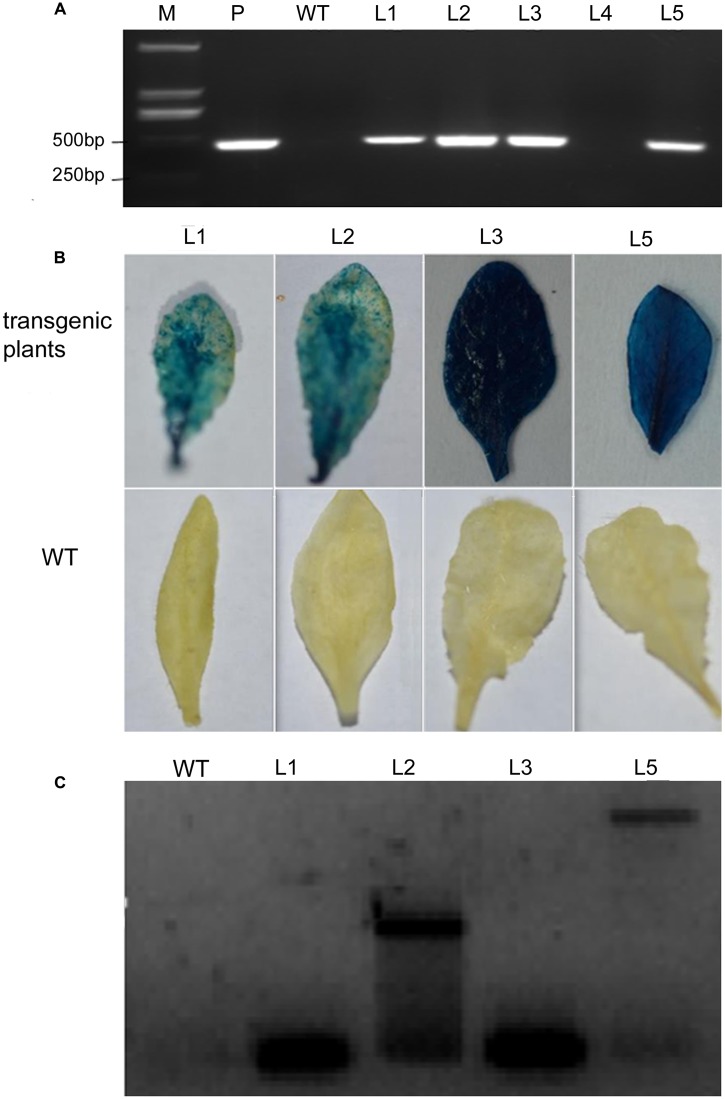
**Detection of transgenic *Arabidopsis* seedlings via over-expression of *VaERD15* from *Vitis amurensis*.** **(A)** PCR analysis of *VaERD15* in T3 transgenic *Arabidopsis* seedlings. **(B)** Leaves of *VaERD15* positive *Arabidopsis* plants tested by GUS staining, with WT *Arabidopsis* plants as controls. **(C)** Southern blotting was carried out on plants considered positive due to previous detection. M, marker; WT, wild type line; P, plasmid (positive control); Ln, various transgenic lines of *VaERD15*.

*Arabidopsis* plants over-expressing *VaERD15* were subjected to PCR. Five T3 *Arabidopsis* plants that survived on selection medium were used for the PCR experiments, and four expected bands were observed (**Figure 5A**). We also carried out a GUS staining assay. Results showed that transgenic *Arabidopsis* leaves were stained blue (**Figure 5B**), indicating that the *GUS* was expressed in the plant *in vivo*. This demonstrates that the plant genome had successfully integrated the target gene *VaERD15*.

Southern blot analysis was performed using four *VaERD15*-positive lines to further confirm that *VaERD15* had been integrated into the *Arabidopsis* genome. The Southern blot results showed that specific hybridization bands could be clearly observed in all positive lines (**Figure 5C**), but not in the WT plants. The four transgenic lines all had a specific hybridization signal, but the band sizes were not all the same. This dissimilarity indicates that the transgenic *Arabidopsis* plants had different insert sites for *VaERD15* in their genomes.

### Response of *VaERD15* to Cold Treatment

**FIGURE 6:**
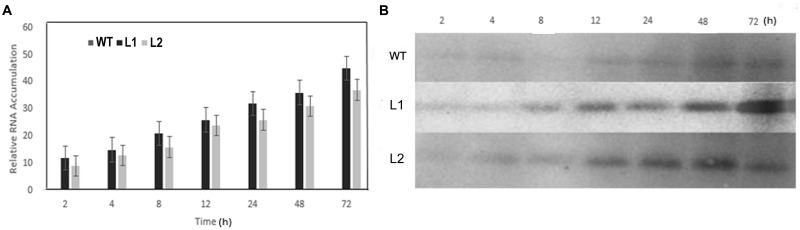
**Assessment of *ERD15* expression levels in *VaERD15-*over-expressed *Arabidopsis* lines at different times of cold stress at -6°C.** **(A)** The relative expression changes of *VaERD15* in WT and two transgenic *Arabidopsis* plants under cold stress. **(B)** Western blotting was carried out to detect expression changes of target protein in *VaERD15*-over-expressing *Arabidopsis* plants induced by cold stress. WT *Arabidopsis* plants were used as controls. All experiments were carried out for three biological replicates.

qRT-PCR and Western blotting were carried out to determine expression changes of *VaERD15 in vivo*. As shown in **Figure 6A**, *VaERD15* transcripts gradually increased in L1 and L2 lines with longer durations of cold stress, but the transcripts were barely detectable in the WT plants. We also detected a change in endogenous *AtERD15* after transferring *VaERD15* into the *Arabidopsis* plant. The qRT-PCR results showed that there were no significant differences in the expression of endogenous *AtERD15* between wild type and transgenic *Arabidopsis*, except that the expression in transgenic plants was nearly four times higher than that in WT *Arabidopsis* at 12 h (**Supplementary Figure S1**). Western blot analysis showed that the *VaERD15* expression in two transgenic *Arabidopsis* lines both tended to increase with longer durations of cold stress, and the *VaERD15* expression level in L1 was higher than that in L2 (**Figure 6B**). However, only weak bands were observed in the WT plants, indicating that expression levels of *VaERD15* in WT and in transgenic *Arabidopsis* were different.

### *VaERD15* Enhanced Cold Tolerance in *Arabidopsis*

**FIGURE 7:**
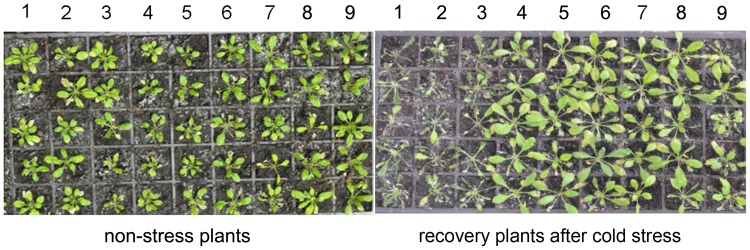
**Phenotypic changes of transgenic and WT *Arabidopsis* lines after 5 days of recovery at room temperature from a cold stress treatment at -6°C.** 1, 2, and 3 are the WT *Arabidopsis* lines; 4, 5, and 6 are the transgenic *Arabidopsis* lines L1; 7, 8, and 9 are the transgenic *Arabidopsis* lines L2.

The cold tolerance assay showed transgenic and WT *Arabidopsis* were both subjected to freezing injury, but to different extents, with longer durations of cold stress. However, freezing injuries were more serious in WT *Arabidopsis*. Almost all the transgenic plants suffering from cold stress were able to resume normal growth after being transferred to room-temperature (∼23°C). However, the same recovery did not occur with the WT plants (**Figure 7**).

Cold tolerance is strongly correlated with a number of physiological parameters in plants (Liu et al., 2010; Li et al., 2014; Xu et al., 2014a). To investigate whether the cold tolerance of *Arabidopsis* lines over-expressing *VaERD15* was improved, we measured several cold-related physiological indices including: relative electrolyte leakage; the contents of proline, MDA; soluble sugars and soluble proteins; and the activities of SOD, POD and CAT.

**FIGURE 8:**
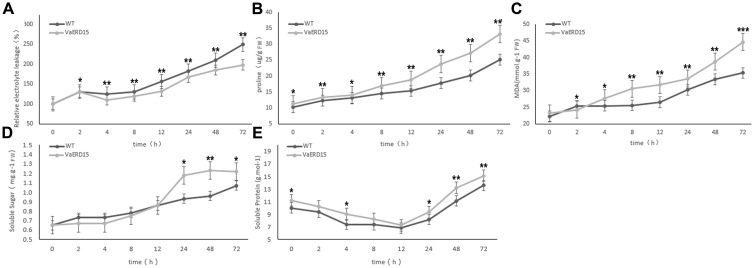
**Assessment of cold-tolerance-related physiological indices in transgenic and WT *Arabidopsis* under cold stress.** The changes of transgenic and WT *Arabidopsis* plants under cold stress in levels of **(A)** relative electrolyte leakage, **(B)** proline, **(C)** MDA, **(D)** soluble sugar and **(E)** soluble protein. Similarly-growing robust transgenic and WT 3-week-old *Arabidopsis* plants were placed at -6°C for 72 h. Leaf samples were collected at 0, 2, 4, 8, 12, 24, 48, and 72 h to assess the relative electrolyte leakage, proline, MDA, soluble sugars and soluble proteins. All determinations were carried out for three biological replicates. Asterisks indicate a significant difference (^∗^*P* < 0.05; ^∗∗^*P* < 0.01) compared with the WT *Arabidopsis.*

Results showed that relative electrolyte leakage in both transgenic and WT *Arabidopsis* plants showed an upward trend with longer durations of low temperature treatment (**Figure 8A**). Both transgenic and WT *Arabidopsis* plants showed similar conductivities in the early stages of cold stress but after 2 h, the conductivity in the WT plants was significantly higher than that in the transgenic plants. This indicates that WT plants suffered greater cell membrane damage than the transgenic plants.

We also investigated proline and MDA contents (**Figures 8B,C**). At the start of the stress period, the proline content of the transgenic plants was slightly higher than that of the WT plants but the difference was not significant. After 4 h of cold stress, proline in the transgenic plants accumulated rapidly, and proline abundance and growth rate were both significantly higher than in the WT plants. Changes in proline content were consistent with the phenotypic changes under cold stress. While the MDA content in the transgenic plants was lower than in the WT plants after 2 h of cold stress, it increased rapidly with longer durations of cold treatment and was consistently higher than in the WT plants.

Similar to conductivity, the soluble sugar contents of both transgenic and WT *Arabidopsis* plants increased with longer durations of cold treatment (**Figure 8D**). The soluble sugar content of the transgenic plants was slightly lower than in the WT plants for the first 12 h of cold stress, but then accumulated rapidly to become significantly higher than in the WT plants.

As shown in **Figure 8E**, the soluble protein content of the transgenic plants was consistently higher than that of the WT plants during cold stress. However, the pattern of change was very similar in both plant, falling to a minimum during the first 12 h, and then gradually increasing.

**FIGURE 9:**
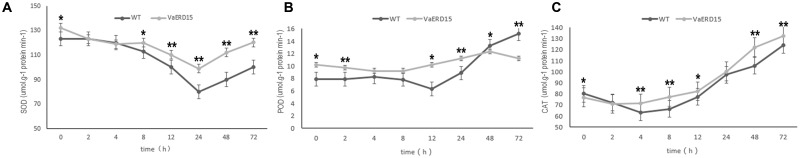
**Assessment of the activity of antioxidant enzymes in transgenic and WT *Arabidopsis* plants under cold stress.** Changes in **(A)** SOD, **(B)** POD, **(C)** CAT activities in transgenic and WT *Arabidopsis* shoots after being subjected to cold stress. Similarly-growing robust transgenic and WT 3-week-old *Arabidopsis* plants were exposed to -6°C for 72 h. Leaf samples were collected at 0, 2, 4, 8, 12, 24, 48, and 72 h to determine the activities of SOD, POD and CAT. All determinations were carried out for three biological replicates. Asterisks indicate a significant difference (^∗^*P* < 0.05; ^∗∗^*P* < 0.01) compared with the WT *Arabidopsis.*

We also measured the activity of antioxidant enzymes. The results showed that SOD activity in transgenic *Arabidopsis* was consistently higher than in WT plants (**Figure 9A**), indicating that plants over-expressing *VaERD15* generally had elevated SOD activity. POD and CAT activities showed a late increase. The POD content curve of the transgenic plants was relatively flat, but that of the WT plants decreased over the first 8 h and then increased significantly after 12 h (**Figure 9B**). The CAT content in the transgenic plants was lower than in the WT plants at first, but rose slightly after 2 h (**Figure 9C**).

## Discussion

Grapes are of considerable economic importance and are grown over large areas of the world. However, close to the cooler limits of where this crop can be grown in the higher latitudes, both north and south of the equator, and at higher altitudes, chilling and frost damage can result in major economic loss. Therefore, the study of genes relating to cold tolerance is crucially important.

*ERD* genes were first isolated from *Arabidopsis* suffering from drought stress (Kiyosue et al., 1994). A large number of studies have shown that over-expressing *ERD* genes can improve the ability of plants to withstand biotic and abiotic stresses. AtERD10 and AtERD14 proteins have been reported to interact with phospholipid vesicles and protect membranes during conditions of high salinity, drought, and low temperature stress (Kovacs et al., 2008). *Brassica juncea ERD4* encoding a RNA-binding protein can respond to the induction of dehydration, ABA, salicylic acid, sodium chloride, cold and heat treatments, and overexpressing *BjERD4* can improve the tolerance of *Arabidopsis* plants to salt and dehydration stresses (Rai et al., 2015). Overexpressing *Arabidopsis ERD10* can activate *CBF/DREB1* genes and enhance the tolerance of plants to cold stress, while a T-DNA insertion mutant of *ERD10* is more sensitive than WT plants to cold stress (Kim and Nam, 2010). However, studies involving *ERD15* and responses to cold stress are few. In our study, a putative transcription factor *VaERD15*, was isolated from a cDNA library of *V. amurensis* induced by low temperatures, and its major function in cold tolerance was investigated.

Our results indicate that *VaERD15* is expressed in diverse plant tissues, which suggests that *VaERD15* is not specific to grapevine. Semi-quantitative PCR results show that accumulation of *VaERD15* transcripts in stems is significantly higher than in roots or leaves after 0 h of chilling. Similar research has shown that transcript accumulation of *SpERD15* under non-stress conditions is higher in roots and old leaves of tobacco (Ziaf et al., 2011). Increasing the duration of cold stress led to down-regulation in the expression of the desired gene in stems, with a concomitant increase in the expression of the gene in leaves and roots. As the expression patterns of *VaERD15* in leaves, roots and stems were different, we speculated that after low-temperature treatment of ‘Heilongjiang seedling’, *VaERD15* could be involved in different regulatory pathways. The study by Yan et al. (2006) analyzed mRNA and protein level expression of 44 genes from *Oryza sativa*, and of the 27 up-regulated genes at the protein level, only five were up-regulated at a transcriptional level due to low-temperature treatment. Baginsky et al. (2005) also found that the correlation in expression at the mRNA and protein levels was lower when *Arabidopsis* chloroplasts and pollen were studied. This phenomenon, i.e., asynchrony in transcription and translation, can be caused by post-translational modifications in the protein-expression process, as well as by operator error (Baldi and Long, 2001). These previous reports support the idea that the expressions of *VaERD15* at the mRNA and protein levels are not identical.

Alves et al. (2011b) suggested that *GmERD15*, as a transcription factor localized to the nucleus and cytoplasm, can activate N-rich protein (*NPR*) gene expression under osmotic stress. *GmERD15* can specifically bind to a 187 bp fragment of *NPR-B* promoter in yeast, and activate the expression of downstream genes. Ziaf et al. (2011) reported that *SpERD15*, located mainly in the nucleus, could enhance the plant’s ability to resist external stress by increasing the accumulation of intracellular solutes and by inhibiting lipid peroxidation. In this study, the analysis of subcellular localization in onion epidermis showed that *VaERD15* was mainly located in the nucleus. Transcription activation experiments confirmed that *VaERD15* was able to activate reporter genes in yeast. Therefore, these results show that *VaERD15* acts as a transcription factor.

To verify the function of *VaERD15* in mitigating cold stress, *Arabidopsis* plants over-expressing *VaERD15* were generated. A survival assay indicated that *Arabidopsis* plants over-expressing *VaERD15* survived better than WT plants under cold stress. We measured the expression levels of the *VaERD15* gene in transgenic *Arabidopsis* and WT plants and the results showed that the transcripts of *VaERD15* increased dramatically with longer durations of cold stress in transgenic *Arabidopsis* compared with WT plants. This observation is consistent with a number of other studies of cold-related genes in transformed plants (Park et al., 2010; Checker et al., 2012; Kidokoro et al., 2015). In addition, qRT-PCR results showed that the expression levels of endogenous *AtERD15* had a slight upward trend in WT plants with increasing duration of cold stress, while the expression of *AtERD15* showed a peak at 12 h in transgenic *Arabidopsis*, which indicated that the introduction of *VaERD15* could enhance the expression of endogenous *AtERD15*. These results illustrate that these transgenic plants are more tolerant to low temperatures. Further investigation indicated that transgenic *Arabidopsis* over-expressing *VaERD15* also showed improved cold tolerance.

Physiological assessment showed cell concentrations of proline, MDA, soluble sugars and proteins, were higher in the over-expressing plants than in the WT plants, especially in the later stages of cold stress. These solutes act in different ways to mitigate stress, including protection of cellular structures, scavenging of ROS and detoxification of enzymes (Xiong et al., 2002; Verma and Dubey, 2003). It has been shown that increases in proline expression under drought, cold and salt stresses can help to protect plants from damage (Wanner and Junttila, 1999; Trovato et al., 2008; Xu et al., 2014b). In our study, rapid increases in proline occurred in transgenic plants after 4 h of cold stress, and after 72 h, proline was 1.5-times higher than in the WT plants. Many studies have reported similar results in plants over-expressing *VaICE1* or *VaICE2*, *OsCOIN*, *OsDREB1* or *DREB1*, *Osmyb4* under cold stress (Ito et al., 2006; Liu et al., 2007; Pasquali et al., 2008; Xu et al., 2014a). Proline accumulation in transgenic plants has been widely reported (Gao et al., 2011; Movahedi et al., 2015; Zhou et al., 2015). Moderate proline accumulations have been observed in transgenic tobacco plants over-expressing *SoMYB18* and a tendency for proline to decrease after cold stress has ceased has also been reported. These inconsistent results may be due to the observation that free proline is not a unique physiological index of osmotic potential reduction (Mao et al., 2011).

Electrolyte leakage is a key indicator of membrane injury caused by stress (Dexter et al., 1932; Jaglo-Ottosen et al., 1998). In the early stages, our *VaERD15-*over-expressing plants and control plants were both able to tolerate cold stress even though their conductivity levels increased. However, under more prolonged stress, the conductivity in the control plants was higher than that in the transgenic plants, indicating greater damage in the controls. The study by Parvanova et al. (2004) was consistent with our results and supported the idea that increased conductivity is indicative of membrane dysfunction.

Malondialdehyde is an end product of lipid peroxidation, and its level is therefore a key indicator of cold stress injury in plants (Jouve et al., 1993; Zhang and Kirkham, 1994). Previous studies on over-expression of *OsAPXa*, *SpERD15* in citrus, rice and tobacco plants reported that, compared with controls, lower levels of MDA were found in transgenic plants after exposure to cold stress (Hara et al., 2003; Sato et al., 2011; Ziaf et al., 2011). In contrast with the above, our results showed that after 2 h of being subjected to cold temperatures, the MDA content of transgenic plants was consistently higher than that of WT plants. However, a similar finding to ours was reported for transgenic tobacco by Parvanova et al. (2004); their results suggested plants develop stress tolerance by producing large amounts of MDA.

Reactive oxygen species accumulation can lead to membrane peroxidation and thus destroy cell structure and function (Mittler et al., 2004). One way in which plants respond to stress is to accelerate free radical scavenging by increasing the activity of protective enzymes. The activities of three antioxidant-related enzymes were measured in our assay. In general, POD activity increased slowly, CAT activity increased rapidly while SOD activity initially increased, then decreased. The time courses of the activity trends for SOD and CAT in our transgenic plants were generally similar to those in WT plants, but the enzyme activities were significantly higher in the transgenic plants. Many reports on environmental stress have recorded the activities of antioxidant enzymes. For example, Yang et al. (2012) confirmed that the activities of POD, SOD and CAT increased in plants over-expressing *OsMYB2*, under salt stress. However, Yuan et al. (2015) reported that under cold stress, antioxidase activity was not significantly different in *VaPAT1-*over-expressing plants compared to WT plants. Shingote et al. (2015) found POD and CAT activities fell, while SOD activity increased in transgenic plants under cold stress. This indicates there may be synergy between the various antioxidant enzymes in the presence of active oxygen scavenging. Therefore, we hypothesize that within 24 h of the imposition of cold stress, the continuing decline in SOD activity that we observed was the result of over-accumulation of CAT in the cell.

We found that in *V. amurensis* the transcription factor *VaERD15* can significantly improve the tolerance of plants to low temperatures. Previous reports have shown that over-expression of individual genes can improve tolerance to cold stress (Saijo et al., 2000; Mukhopadhyay et al., 2004; Mishra et al., 2013). Other studies have also indicated that transformation of cold-resistant genes can activate and enhance the expression of related genes under cold stress (Ziaf et al., 2011; Xu et al., 2014a). Therefore, clarifying the interaction between *VaERD15* and other genes appears to be a promising direction for future research in this field. In summary, our findings confirm the significant value of continued investigation into the function and mechanisms of *ERD* genes in grapes, for the further development of cold-tolerant strains.

## Author Contributions

DY: Expression and analysis of *VaERD15* in *Arabidopsis*, determining the physiological and biochemical indices for transgenic plants. LZ: RT-PCR analysis of *AtERD15*, data collation and manuscript writing. KZ: Subcellular localization of VaERD15 and transcriptional activation analysis of *VaERD15*. RN: Cloning and sequence analysis of *VaERD15* gene, as well as RT-PCR analysis for *V. amurensis*. HZ: Semi-quantitative RT-PCR analysis for *V. amurensis*. JZ: Experimental design, plant material preparation and manuscript modification.

## Conflict of Interest Statement

The authors declare that the research was conducted in the absence of any commercial or financial relationships that could be construed as a potential conflict of interest.

## Footnotes

1 http://www.ncbi.nlm.nih.gov/gorf/gorf.html

2 http://prosite.expasy.org/

3 http://www.megasoftware.net

## Supplementary Material

**FIGURE S1 | The relative expression changes of *VaERD15* and *AtERD15* in transgenic and WT *Arabidopsis* plants under cold stress.** All experiments were carried out for three biological replicates.

## Abbreviations

*AtGAPDH*: *Arabidopsis thaliana* glyceraldehyde 3-phosphate dehydrogenase
CAT: catalase
*CBF*: C-repeat-binding factor
cDNA: complementary DNA
*DREB1*: dehydration responsive element-binding factor 1
*ERD*: early responsive to dehydration
EST: expressed sequence tag
GFP: green fluorescent protein
GUS: β-glucuronidase
MDA: malondialdehyde
MS: Murashige and Skoog
NCBI: National Center for Biotechnology Information
ORF: open reading frame
PCR: polymerase chain reaction
POD: peroxidase
qRT-PCR: real-time quantitative polymerase chain reaction
ROS: reactive oxygen species
SOD: superoxide dismutase
WT: wild type
