# Supplementary figures and images for: VaERD15, a Transcription Factor Gene Associated with Cold-Tolerance in Chinese Wild Vitis amurensis
Source: Front Plant Sci. 2017 Mar 7;8:297. doi: 10.3389/fpls.2017.00297 (PMC5339311; doi:10.3389/fpls.2017.00297)

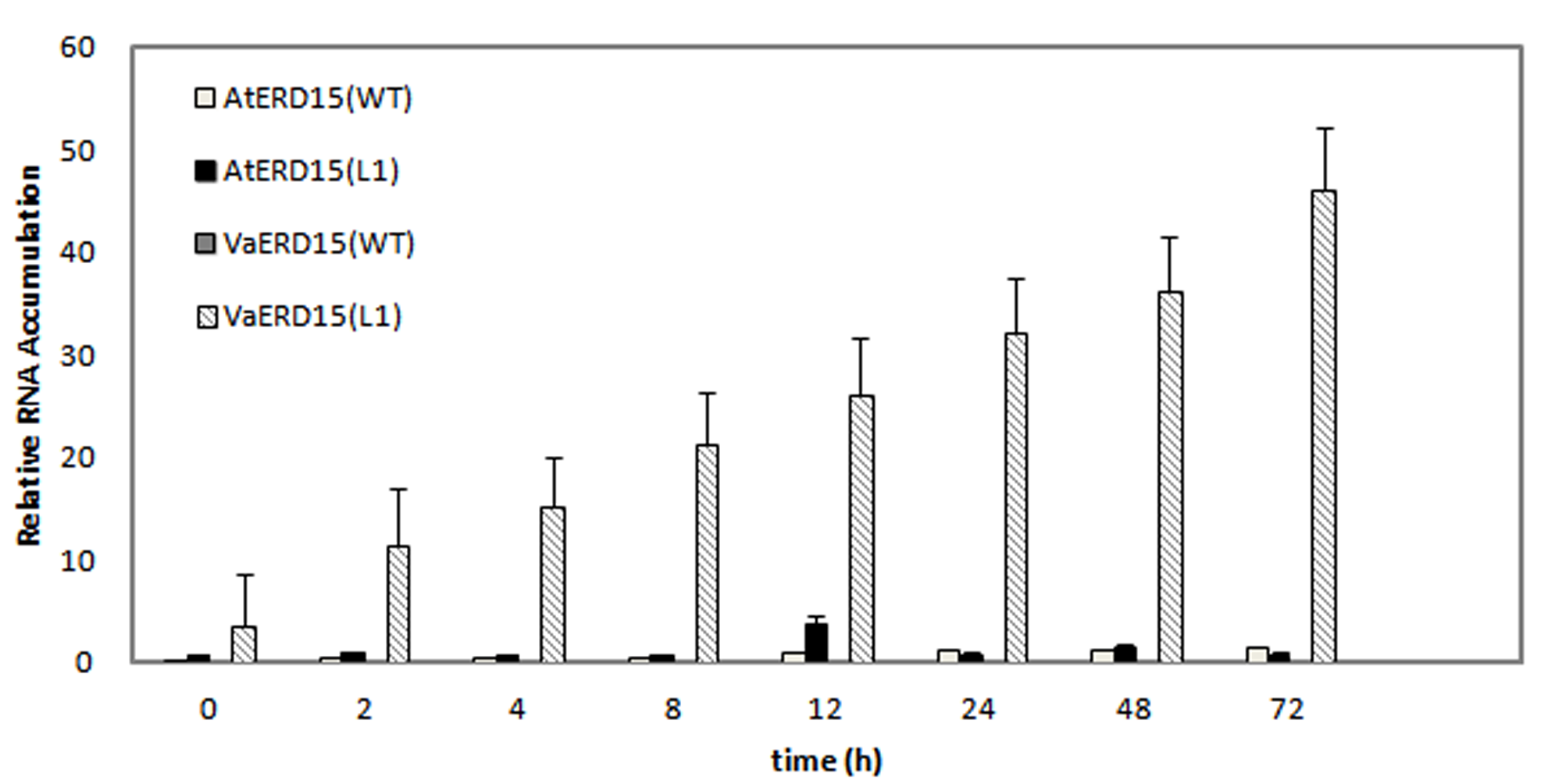

Supplement: Supplementary file 1 [file Image_1.TIF]
